# Effects of Remimazolam versus Sevoflurane on Hemodynamics in Patients Undergoing Coil Embolization of Cerebral Aneurysm: A Prospective Randomized Controlled Trial

**DOI:** 10.3390/jcm13133958

**Published:** 2024-07-06

**Authors:** Eunji Ko, Lee Gyeong Je, Jang Hun Kim, Yeon Jae Song, Choon Hak Lim

**Affiliations:** 1 Department of Anesthesiology and Pain Medicine, Korea University Anam Hospital, Seoul 02841, Republic of Korea; froken.ko@gmail.com (E.K.); jellyg0209@gmail.com (L.G.J.); jennysong1997@gmail.com (Y.J.S.); 2 Department of Neurosurgery, Korea University Anam Hospital, Korea University College of Medicine, Seoul 02841, Republic of Korea; jhkimns@naver.com; 3 Department of Anesthesiology and Pain Medicine, College of Medicine, Korea University, Seoul 02841, Republic of Korea

**Keywords:** blood pressure monitors, general anesthesia, intracranial aneurysm, therapeutic embolization

## Abstract

**Background:** Cerebral aneurysm coil embolization is often performed under general anesthesia to prevent patient movement and sudden high blood pressure. However, the optimal anesthetic agent remains uncertain. This study aimed to determine whether maintaining anesthesia with remimazolam in patients undergoing coil embolization could avoid hypotension or hypertension compared to sevoflurane. **Methods:** Thirty-three adult patients participated in this single-blinded, randomized controlled trial. Patients in Group R were induced and maintained with remimazolam, whereas those in Group S received propofol and sevoflurane. **Results:** The use of remimazolam significantly reduced the incidence of intraoperative hypotension events (33.3% vs. 80.0%; *p* = 0.010) but did not change the incidence of hypertension events (66.7% vs. 73.3%; *p* = 0.690). Patients in Group R maintained a significantly higher range of maximal (100.2 ± 16.6 vs. 88.1 ± 13.5 mmHg; *p* = 0.037) and minimal (69.4 ± 6.6 vs. 63.4 ± 4.8 mmHg; *p* = 0.008) mean arterial blood pressure than those in Group S during the intervention. **Conclusions:** This is the first study to demonstrate the feasibility of maintaining general anesthesia with remimazolam in patients undergoing cerebral aneurysm coil embolization. The findings suggest that remimazolam may maintains better hemodynamic stability, reducing the incidence of hypotensive events without compromising patient safety.

## 1. Introduction

Intracranial aneurysms are a common condition affecting approximately 1–2% of the population [1]. Unruptured cerebral aneurysms must be managed with consideration of both short-term and long-term risks, because the rupture of cerebral aneurysm can cause subarachnoid hemorrhage, posing a significant medical burden to patients. Over the decades, several treatments have been researched, and coil embolization, a minimal-invasive endovascular intervention, has shown favorable outcomes [2]. Since aneurysms can rupture even with the slightest movement during the procedure, general anesthesia with neuromuscular blockade is maintained using various methods, such as inhalation and intravenous anesthesia [3]. However, the optimal anesthetic approach has not been confirmed [4,5]. The important thing is to prevent excessive fluctuations in blood pressure during intervention. A sudden peak in blood pressure can cause the rupture of cerebral aneurysm [3], while anesthesia-induced hypotension may increase the risk of cerebral infarction after surgery [6].

Remimazolam is an ultra-short-acting benzodiazepine that can be used to sedate patients without respiratory depression during procedures that require sedation. By adjusting the dose, effective anesthetic depth can be achieved for general anesthesia [7]. The most notable advantage of remimazolam as a general anesthetic agent is its lower cardiovascular depressant effect than that of propofol or inhalation anesthetic agents [7,8,9]. For this reason, its usefulness has been demonstrated through research in older adult patients, patients with a high American Society of Anesthesiologists physical status class [10], and patients who underwent cardiovascular surgery [9]. However, its superiority in neurosurgery remains unproven due to limited usage duration [11].

Sevoflurane causes the least increase in intracranial pressure among inhalation anesthetics [12], and sevoflurane with remifentanil is used during neurosurgeries that do not involve neuromonitoring. Its combined use helps prevent blood pressure from soaring by reducing excessive stimulation such as tracheal intubation [13]. However, because there is no strong pain sensation during coil embolization, inhalation anesthesia with remifentanil is likely to cause prolonged hypotension. Additional vasopressors are often required to prevent limited oxygen supply to organs, including cerebral infarction.

This study hypothesized that remimazolam could help maintain appropriate blood pressure and aimed to determine whether maintaining anesthesia with continuous infusion of remimazolam in patients undergoing coil embolization for cerebral aneurysms could prevent hypotension compared to using an inhaled anesthetic (sevoflurane). Substitution with remimazolam may significantly attenuate sevoflurane-induced hypotensive events. However, remimazolam may not prevent excessive increases in blood pressure that could be effectively controlled by sevoflurane. This may increase the risk of rupture of cerebral aneurysms. If the changes in blood pressure over time during surgery in the group using remimazolam and the group using sevoflurane are compared, and it is confirmed which anesthetic drug more easily controls blood pressure, it will be helpful to decide which drug to use as the first-line drug for interventional neurosurgical procedures.

## 2. Materials and Methods

This prospective, single-blinded, randomized controlled trial was approved by the Institutional Review Board of Korea University Anam Hospital (IRB No. 2023AN0097, approval date: 2 March 2023) and registered with Clinical Research Information Service (CRIS, http://cris.nih.go.kr; KCT0008343, registration date: 7 April 2023). Adults aged over 19 years undergoing coil embolization of unruptured cerebral aneurysm under elective general anesthesia in the Korea University Anam Hospital were selected for consent to the study. Patients who have already been intubated and sedated before general anesthesia; patients in whom the decision for intubation was made after coil embolization; patients who are hemodynamically unstable due to shock or coma; patients with hypersensitivity or idiosyncratic reactions to anesthetic drugs including sevoflurane, remifentanil, or benzodiazepine drugs; patients with absolute contraindications to the administration of remimazolam (patients with acute narrow-angle glaucoma, acute alcoholism with suppressed vital signs, severe or acute respiratory failure, genetic lactose intolerance such as galactose intolerance, and severe hypersensitivity to Dextran 40); patients who have difficulty completing the consent form or who do not consent to the study; patients who are vulnerable to the study; and patients who could not monitor arterial blood pressure continuously through the radial artery were excluded from the study.

As there have been no studies on the use of remimazolam in coil embolization of cerebral aneurysms, preliminary research was necessary to calculate the target number of subjects. In a similar study, Liu et al. observed hemodynamic changes with remimazolam during valve replacement surgery and recruited 15 patients each in the control and experimental group [9]. Thirty patients were recruited in Hasegawa’s study, which reported pulse changes with remimazolam [14]. Initially, researchers planned to recruit eight patients per group through a pilot study. Twenty-five percent of the eight patients who used remimazolam experienced intraoperative hypotension, while 75% of the patients who maintained the existing anesthesia method experienced intraoperative hypotension. G*Power was utilized for a two-tailed z-test to assess the difference between control (75%) and experimental (25%) group proportions. With a significance level (α) of 0.05 and 80% power, a sample size of 30 (15 per group) was determined, achieving an actual power of 81.57% (G*Power version 3.1.9.7, Heinrich-Heine-Universität Düsseldorf, Germany). Considering a dropout rate of 10%, the final sample size was determined to be 33 patients.

Informed consent was obtained before the surgery from patients who met the inclusion criteria but did not meet the exclusion criteria. After the patient entered the operating room on the day of surgery, the criteria and consent were checked. The patients were randomly assigned to either the experimental group or the control group at a ratio of 1:1 by the corresponding researcher using computer-generated randomization according to a table using www.randomization.com. The results of the randomization were sealed in an opaque envelope, blinded to researchers and patients other than the corresponding researcher, and disclosed during data analysis after the study was completed.

In the experimental group (Group R), anesthesia was induced and maintained with remimazolam (Byfavo, Hana Pharm, Republic of Korea). The drug was diluted to 1 mg/mL and initially infused at a rate of 6–12 mg/kg/h. Following this, the high infusion rate of remimazolam was maintained for 5 to 10 min during induction, and an additional neuromuscular blocking agent and remifentanil or nicardipine were injected before intubation. After intubation, anesthesia was maintained by infusing remimazolam at 1–2 mg/kg/h. It is mainly maintained at 1 mg/kg/h, but higher doses can be injected when necessary.

In the control group (Group S), anesthesia was induced by a bolus injection of 1–2.5 mg/kg propofol and maintained with sevoflurane at a concentration of 1.0 minimum alveolar concentration (MAC).

All interventions were performed by a single surgeon (J.H.K.) who was blinded to treatment allocation. All anesthesia was performed under the supervision of the corresponding researcher (E.K.). All patients did not receive pre-medication before anesthesia, including preoperative sedatives or anticholinergics. Patients admitted to the operating room had lidocaine applied to the skin around the radial artery and were placed under local anesthesia and fitted with an intra-arterial blood pressure monitoring device. After inducing anesthesia in each group, rocuronium, a neuromuscular blocking agent, was injected at a bolus dose of 0.6 to 1.0 mg/kg. To attenuate sudden stimulation, tracheal intubation was performed after a bolus injection of 0.5–1.0 mcg/kg/min of remifentanil or an appropriate amount of nicardipine. After intubation, anesthesia was maintained using the drugs designated for each group. Remifentanil was maintained at an initial basal concentration of 0.05 mcg/kg/min.

Patients admitted to the operating room were placed on intra-arterial blood pressure monitoring devices under local anesthesia with lidocaine on the skin around the radial artery. After inducing anesthesia with a setting in each group, a neuromuscular blocking agent, rocuronium, was injected as a bolus dose of 0.6–1.0 mg/kg. To attenuate the sudden stimulation, tracheal intubation was performed after a bolus injection of 0.5–1.0 mcg/kg/min of remifentanil or an appropriate amount of nicardipine. After intubation, anesthesia was maintained using the medication specified for each group. Remifentanil was maintained at an initial basal concentration of 0.05 mcg/kg/min.

The primary outcome was the occurrence of hypotension or hypertension during surgery. When the events occur, vasopressors or vasodilators were administered, and the amount and number of medications were recorded. The secondary endpoints were the patient’s intraoperative blood pressure and pulse; patient mortality within 30 days after surgery; and the incidence rate of postoperative complications, including postoperative delirium (POD) and acute kidney injury (AKI) within postoperative 7 days (according to the Kidney Disease Improving Global Outcomes [KDIGO]). During the intervention, systolic arterial blood pressure (SABP), diastolic arterial blood pressure (DABP), and mean arterial blood pressure (MABP) were automatically recorded in electronic medical records (EMR) every 5 min. Among all collected data, the highest and lowest SABP, DABP, and MABP values during anesthesia were analyzed. Vital signs were also analyzed at specific time points during anesthesia: initiation of anesthesia (anesthesia start), before intubation, after intubation, start of surgery (operation start), end of surgery (operation end), after extubation, and termination of anesthesia (anesthesia end).

The optimal range of blood pressure during the intervention was considered to be up to 20% above and 20% below the blood pressure at the initiation of anesthesia. If the MABP fell below 65 mmHg or the optimal range for 60 s, this was termed a hypotension event. Vasopressors such as ephedrine, phenylephrine, norepinephrine, and epinephrine were injected. Remifentanil was maintained even if a single vasopressor bolus was administered during the patient’s initial hypotensive event. When hypotension events repeated more than twice, continuous infusion of vasopressors began. At this time, remifentanil infusion was discontinued. On the contrary, events in which the MABP was higher than the optimal blood pressure range for 60 s were called hypertension events. If the patient developed a hypertensive event, the infused rate of remifentanil was increased to 0.10 mcg/kg/min and a nicardipine bolus of 0.2 to 0.5 mg was injected. For persistent hypertensive events, the remifentanil rate was increased to 0.15 or 0.20 mcg/kg/min. If hypertension events were not controlled, nicardipine infusion was started. Other antihypertensive drugs (esmolol or labetalol) were administered if needed. The types and amounts of the vasoactive agents used were all recorded. Intraoperative heart rate (HR) and mortality within 30 days after surgery, the incidence rate of POD within 7 days after surgery [15], and postoperative AKI (based on KDIGO guidelines) were collected by a co-researcher who was blinded to the EMR and case reports 30 days after the intervention.

**Figure 1:**
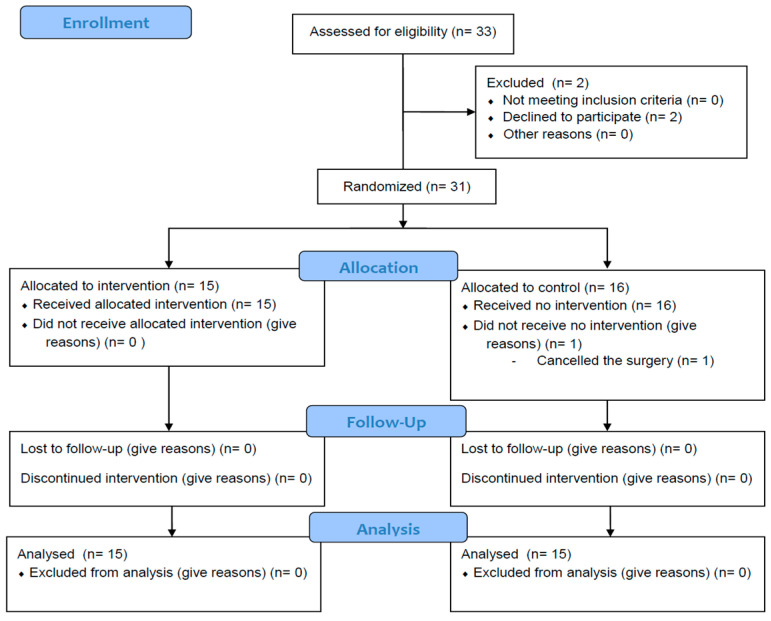
CONSORT flow diagram.

The collected data were subjected to statistical analysis using SPSS software (SPSS version 27.0, IBM, Chicago, IL, USA) at the Department of Medical Statistics, College of Medicine, Korea University, which was not related to this study. Continuous variables between the two groups were compared using Student’s *t*-test or Mann–Whitney U-test. Normally distributed data were analyzed using Student’s *t*-test, and non-normally distributed data were analyzed using the Mann–Whitney U-test. The chi-squared test was used to determine the frequency of postoperative complications. All data were expressed as mean ± standard deviation, median (first quartile, third quartile), or number of patients (%). *p*-value was reported as a two-tailed value, and *p* < 0.05 was considered to be statistically significant. This manuscript adheres to the applicable CONSORT guidelines (Figure 1).

## 3. Results

From 27 March 2023 to 22 November 2023, thirty-three patients were initially enrolled in this study. Two patients withdrew consent before surgery. One patient in Group S was excluded due to hemodynamic instability on the morning of surgery. Finally, 30 patients participated (Figure 1). The patient characteristics are shown in Table 1. Patients in Group R had significantly more cerebral aneurysms in the basilar artery than patients in Group S. Besides this, all demographic data and characteristics of the patients in Groups B and S showed no significant differences. Patients in Group R received 1.56 ± 0.29 mg/kg/h of remimazolam. Among the distribution of injected remimazolam infusion rate, the first quartile was 1.45 mg/kg/h and the third quartile was 1.73 mg/kg/h.

### 3.1. Primary Outcomes

#### Hypotension or Hypertension Events during Intervention

The distribution of patients who experienced hypotension and hypertension during surgery is shown in Table 2. The incidence of hypotensive events during the intervention was significantly higher in Group S (*p* = 0.010). Five patients in Group R (33.3%) and 12 patients in Group S (80.0%) developed hypotension. The number of patients who required continuous infusion of vasopressors to maintain optimal blood pressure was also significantly more in Group S (11 patients (73.3%); *p* = 0.001) than in Group R (2 patients (13.3%)). According to the type of vasopressor, the amount of phenylephrine used was more in Group S than in Group R (*p* < 0.001). In the case of ephedrine, the average usage was higher in Group S, but the difference was not significant (*p* = 0.268). None of the patients were administered norepinephrine or epinephrine. The incidence of hypertensive events during surgery was not significantly different between Groups B and S (*p* = 0.690). Only nicardipine was used as a vasodilator. Continuous infusion of the vasodilator was not performed, and there was no difference in the number of vasodilators administered between the two groups.

### 3.2. Secondary Outcomes

#### 3.2.1. Intraoperative Blood Pressure and Pulse

**Figure 2:**
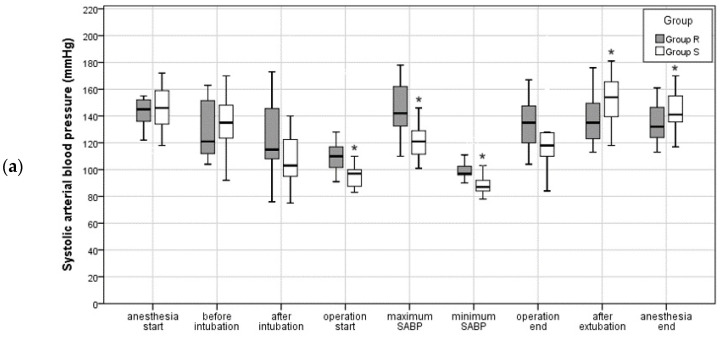

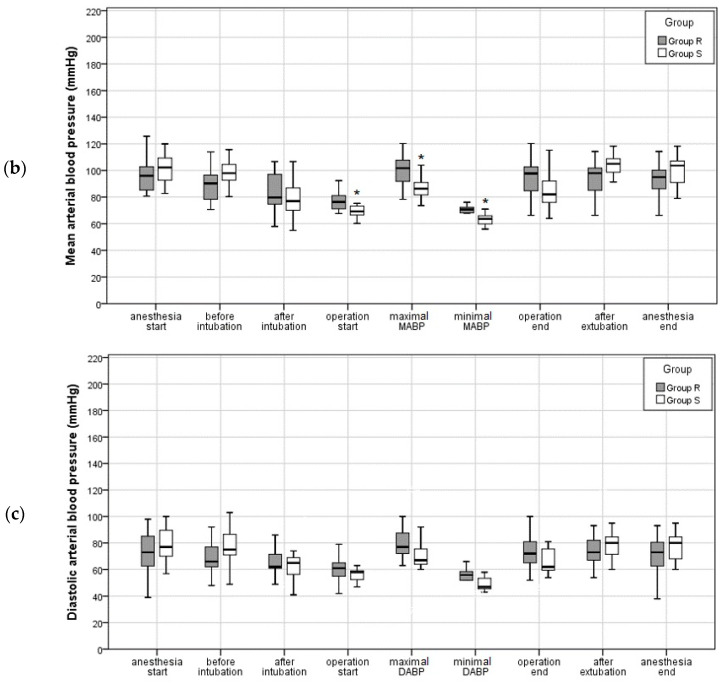
Perioperative arterial blood pressure (mmHg) during general anesthesia: (**a**) systolic arterial blood pressure; (**b**) median arterial blood pressure; (**c**) diastolic arterial blood pressure. If there is a significant difference (*p* < 0.05) in the distribution of blood pressure between Group R and group S, it is marked with asterisk (*).

The patients’ blood pressure (BP) during the intervention is displayed in Figure 2. The graphs are error bar plots, where the upper and lower bars represent the 95% confidence interval, and the center denotes the mean value. In Group R, maximal and minimal SABP were 146.0 ± 20.0 and 99.6 ± 6.8 mmHg, respectively. Maximal SABP in Group S was 124.6 ± 21.4 mmHg, and minimal SABP was 88.4 ± 8.1 mmHg. Compared to Group S, the maximal and minimal SABP of Group R were significantly higher (*p* = 0.009 and *p* < 0.001, respectively) (Figure 2a). The distribution of maximal and minimal MABP (100.20 ± 16.56 mmHg and 69.38 ± 6.57 mmHg, respectively) in Group R was also significantly higher than that (88.08 ± 13.55 mmHg and 63.41 ± 4.80 mmHg, respectively) in Group S (*p* = 0.037 and *p* = 0.008, respectively) (Figure 2b). However, there was no significant difference in maximal and minimal DABP between Group R (78.20 ± 17.56 mmHg and 53.73 ± 9.11 mmHg, respectively) and Group S (70.20 ± 12.16 mmHg and 49.53 ± 4.85 mmHg, respectively) (*p* = 0.158 and *p* = 0.126, respectively) (Figure 2c).

At the operation start, SABP and MABP in Group R (112.2 ± 17.8 and 77.8 ± 9.97 mmHg, retrospectively) were significantly higher than that in Group S (95.5 ± 8.8 and 69.2 ± 5.2 mmHg, retrospectively) (*p* = 0.003 and *p* = 0.006, retrospectively) (Figure 2). After extubation, SABP in Group R was significantly lower than that in Group S (138.3 ± 18.3 vs. 152.1 ± 18.3 mmHg; *p* = 0.048). At the anesthesia end, the SABP of in Group R was relevantly lower than that in Group S (134.2 ± 13.9 vs. 145.1 ± 16.5 mmHg; *p* = 0.060).

**Figure 3:**
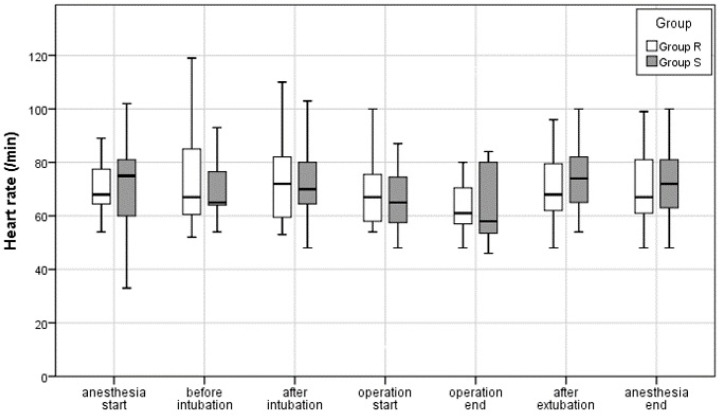
Perioperative heart rate (/min) during general anesthesia.

There were no significant differences in HR at any time point between Group R and Group S (Figure 3).

#### 3.2.2. Postoperative Complications

Each group experienced one event during the study period. One patient in Group R showed temporary leakage of contrast in the posterior communicating artery aneurysm, which was resolved. The other single patient in Group S experienced failure of coil embolization for one of the two cerebral aneurysms. In both groups, no patients died within 30 days of the intervention. POD or AKI did not occur in any patient. For one patient in Group S, a single paroxysmal atrial fibrillation event was identified on postoperative day 1 within 24 h after surgery, but no other events occurred thereafter.

## 4. Discussion

This study compared the perioperative vital signs of patients who received continuous remimazolam infusion with those maintained under anesthesia with an inhaled anesthetic (sevoflurane) to determine whether remimazolam helped maintain blood pressure in patients undergoing coil embolization for cerebral aneurysms. Compared to patients who used sevoflurane as the anesthesia maintenance agent, those who maintained anesthesia with remimazolam maintained a higher range of maximal and minimal SABP and maximal and minimal MABP during the intervention. The use of remimazolam significantly reduced the incidence of intraoperative hypotensive events but did not change the incidence of hypertensive events. This study revealed that maintaining anesthesia with remimazolam, rather than sevoflurane, prevents hypotension, attenuates hemodynamic fluctuation, and does not increase the risk of mortality or postoperative complications significantly. In particular, it was inferred that the rate of hypertension decreased after surgery, making it easier to control blood pressure.

Since its approval as a general anesthetic agent in South Korea in 2022, remimazolam use has dramatically increased. The blood concentration peaks in 1–2 min and begins its sedative effect within 1 min [16]. In addition, it is an ultra-short-acting benzodiazepine with an elimination half-life of approximately 45 min, showing a significantly faster elimination rate than that of other benzodiazepines. Remimazolam was initially administered during procedures requiring sedation to preserve airway reflexes and respiratory drive to some extent. Several studies have reported on the safety of respiratory patency [17,18]. However, remimazolam is advantageous as a maintenance drug for general anesthesia because of its excellent hemodynamic stability during the perioperative period. Blunt hemodynamic variability of remimazolam has been reported in many previous studies. These properties may reduce the need for vasoactive drugs [19] and offset perioperative hemodynamic changes in hypertensive patients [20]. This study was initiated with the assumption that hemodynamic safety would be helpful for patients with cerebrovascular diseases and concomitant cardiovascular diseases, including hypertension. These results are consistent with previous studies [21,22].

In this study, there was a significant difference in the amount of rocuronium used between the two groups. To ensure an accurate procedural image and comfort of the intervention, brain awakening status was not monitored by attaching a bispectral index (BIS) to the forehead. Sufficient depth of anesthesia and analgesia are required to ensure patient immobility during the embolization. For patients maintained under general anesthesia using sevoflurane, MAC can be used to confirm sufficient depth of anesthesia. In contrast, in the case of remimazolam, there is no other indicator of depth of anesthesia unless BIS is monitored. Additionally, the anesthesiologist may have been reluctant to increase the dose of remifentanil due to persistent hypotension. This is believed to induce the increased use of rocuronium in the Group R. However, because 200 mg of sugammadex was used uniformly for all included participants, there was no difference in the amount of sugammadex used. No respiratory complications were observed after surgery. In addition, there was no difference in the time required for extubation.

This study had several limitations. First, as mentioned, because the depth of anesthesia was not monitored, it is difficult to guarantee that remimazolam and sevoflurane maintained the same depth of anesthesia. Because the use of anesthetic drugs can induce a decrease in blood pressure in proportion to the dose, it is a prerequisite that the anesthetic drug doses be similar between the two groups. To overcome this, patients in both groups tried to consistently maintain the known dosage for general anesthesia. All patients in Group S maintained sevoflurane concentration at 1.0 MAC as much as possible. Patients in Group R also maintained the dosage known in previous studies [23,24,25]. The anesthesia concentration for most patients was 1 mg/kg/h, and it was confirmed that an average of 1.56 mg/kg/h was injected due to the initial bolus dose. The concentration of remimazolam was not adjusted considering the patient’s blood pressure but was adjusted according to the patient’s movement or surgical needs. However, additional studies implementing the depth of anesthesia for the same surgery will be needed. Additionally, it is difficult to guarantee that remifentanil administered in combination will produce the same level of analgesic effect in both groups. Therefore, additional research that includes not only BIS but also pain monitoring equipment such as analgesic nociception index appears to be necessary. Second, because the primary outcome, that is, intraoperative blood pressure, showed a significant difference between the two groups, the sample size for other outcomes was small. Therefore, although no underlying morbidity showed a significant difference in distribution, the distribution did not completely match. Additionally, the mortality and incidence rates of POD and AKI were 0%. A meaningful comparative analysis of the postoperative complications was not performed. Because the incidence of complications after surgery is low, it is necessary to conduct a large-scale study to demonstrate not only blood pressure but also the risk of clinical mid- to long-term postoperative complications.

Until further studies show that remimazolam significantly improves postoperative outcomes compared to sevoflurane, the use of remimazolam may be economically burdensome. The price of remimazolam is KRW 42,890 (USD 31) per bottle, while propofol and sevoflurane in Group S are cheaper at KRW 3086 (USD 2.23) and KRW 3458 (USD 2.50) per bottle, respectively. The average usage of remimazolam in Group R was 196 mg, and patients in Group R paid an average of KRW 200,000 (USD 150) solely for anesthetic agents. When obtaining consent for this research, researchers informed study subjects that anesthesia fees might be higher if assigned to the experimental group. However, it is assumed that patients in Group S have a high likelihood of incurring additional costs for vasoconstrictors such as phenylephrine (KRW 839, USD 0.61), ephedrine (KRW 377, USD 0.27), or norepinephrine (KRW 1756, USD 1.27). In fact, the amount of phenylephrine used was significantly higher. Considering the economic benefits and losses, further research will be necessary to analyze the postoperative prognosis, long-term complications, and healthcare costs incurred between the two groups.

## 5. Conclusions

This is the first study to apply general anesthesia using remimazolam in patients undergoing coil embolization for cerebral aneurysms. Although additional studies on cerebrovascular events are needed [26], the use of remimazolam appears to prevent low blood pressure, which frequently occurs during interventional procedures with general anesthesia, and is useful even in irritating situations such as intubation and extubation. If positive results are obtained through continuous comparative studies with other drugs, remimazolam may be preferentially used in interventional procedures, including cerebral aneurysm coil embolization.

## Figures and Tables

**Table 1:** Demographic and clinical variables between Groups R and S.

	Group R (*n* = 15)	Group S (*n* = 15)
**Demographics**		
Age	61.4 ± 15.1	60.8 ± 11.7
Sex (male, %)	11 (73.3)	8 (53.3)
ASA-PS class	3 [3, 3]	3 [3, 3]
BMI	23.8 ± 2.6	24.1 ± 2.6
**Cardiac comorbidities**		
Hypertension (%)	11 (73.3)	8 (53.3)
Coronary artery disease (%)	2 (13.3)	2 (13.3)
Arrhythmia (%)	3 (20.0)	1 (6.7)
Cardiomyopathy (%)	1 (6.7)	0 (0.0)
**Pulmonary comorbidities**		
Asthma (%)	1 (6.7)	0 (0.0)
Pneumonia (%)	2 (13.3)	0 (0.0)
Effusion in lung (%)	1 (6.7)	2 (13.3)
Current smoking (%)	3 (20.0)	5 (33.3)
**Other comorbi** **dities**		
Diabetes mellitus (%)	3 (20.0)	3 (20.0)
Hypothyroidism (%)	1 (6.7)	0 (0.0)
Chronic kidney disease (%)	2 (13.3)	1 (6.7)
Cerebral hemorrhage (%)	2 (13.3)	3 (20.0)
Cerebral infarction (%)	2 (13.3)	0 (0.0)
Psychologic disorder (%)	2 (13.3)	4 (26.7)
Parkinson’s disease (%)	0 (0.0)	1 (6.7)
Chronic alcoholic (%)	1 (6.7)	0 (0.0)
**Location of aneurysms**		
Right side (%)	8 (53.3)	6 (40.0)
Left side (%)	6 (40.0)	10 (66.7)
Anterior communicating artery (%)	3 (20.0)	3 (20.0)
Posterior communication artery (%)	1 (6.7)	0 (0.0)
Anterior cerebral artery (%)	2 (13.3)	4 (26.7)
Basilar artery (%)	4 (26.7)	1 (6.7)
Vertebral artery (%)	2 (13.3)	0 (0.0)
Paraclinoid internal carotid artery (%)	3 (20.0)	6 (40.0)
Posterior inferior cerebellar artery (%)	0 (0.0)	1 (6.7)
Internal carotid artery (%)	1 (6.7)	2 (13.3)
**Intraoperative data**		
Duration of intervention (min)	127.5 ± 65.2	103.7 ± 44.2
Time from the end of intervention to extubation (min)	11.1 ± 5.6	12.1 ± 7.4
Duration of anesthesia (min)	185.0 ± 67.4	155.0 ± 38.5
Total infused fluid (mL)	520.0 ± 273.7	560.0 ± 452.8
Total amount of remifentanil (mg)	0.88 ± 0.72	0.64 ± 0.63
**Intraoperative events**		
Intraoperative awakening (%)	0 (0.0)	0 (0.0)
Thromboembolic event (%)	0 (0.0)	0 (0.0)
Hemorrhagic event (%)	0 (0.0)	0 (0.0)
Other intraoperative events (%)	1 (6.7)	1 (6.7)

**Table 2:** Number of patients who experienced intraoperative events of hypotension or hypertension.

	Group R (*n* = 15)	Group S (*n* = 15)	*p*-Value
**Hypotension Events**			
Number of patients who experienced hypotension (%) Number of patients to whom vasoconstrictors infused (%)	5 (33.3)	12 (80.0)	* 0.010
	2 (13.3)	11 (73.3)	* 0.001
Amount of phenylephrine injected (mg)	0.35 ± 1.30	4.35 ± 1.74	* <0.001
Amount of ephedrine injected (mg)	1.07 ± 2.37	2.67 ± 4.94	0.268
Amount of norepinephrine injected (mg)	0.00 ± 0.00	0.00 ± 0.00	1.000
Amount of epinephrine injected (mg)	0.00 ± 0.00	0.00 ± 0.00	1.000
**Hypertension Events**			
Number of patients who experienced hypotension (%) Number of patients to whom vasoconstrictors infused (%)	10 (66.7)	11 (73.3)	0.690
	0 (0.0)	0 (0.0)	1.000
Amount of nicardipine injected (mg)	1.11 ± 1.43	0.73 ± 0.68	0.499
Amount of labetalol injected (mg)	0.00 ± 0.00	0.00 ± 0.00	1.000
Amount of esmolol injected (mg)	0.00 ± 0.00	0.00 ± 0.00	1.000

* The significant difference (*p* < 0.05) between Group R and Group S.

## Data Availability

The data presented in this study are available on request from the corresponding author.
